# Serious Delayed Hair Toe Tourniquet Syndrome with Bone Erosion and Flexor Tendon Lesion

**DOI:** 10.1155/2014/592323

**Published:** 2014-10-14

**Authors:** Nicola Bizzotto, Andrea Sandri, Dario Regis, Guillherme Carpeggiani, Franco Lavini, Bruno Magnan

**Affiliations:** Department of Orthopaedic and Trauma Surgery, Integrated University Hospital, Piazzale A. Stefani 2, 37126 Verona, Italy

## Abstract

Hair toe tourniquet syndrome (HTTS) is an uncommon pediatric condition occurring when the toe is circumferentially strangulated by human hair or fibers. An 8-week-old little girl was admitted to the Emergency Department because of the worsening swelling in the right second and third toes, which had been been previously treated with a local antibiotic thinking of an infection. An unrecognized HTTS was leading the third toe to necrosis. An urgent release of the constricting band on the two toes was performed and bone erosion and partial flexor tendon lesion on the third toe were detected. We would like to raise awareness in the community and in colleagues about HTTS in children, because early recognition and urgent treatment are mandatory to provide an adequate management and prevent severe complications.

## 1. Introduction

Hair toe tourniquet syndrome (HTTS) is an unusual condition that occurs when toe is circumferentially strangulated by a strand of hair, thread, or fiber [1]. HTTS infrequently occurs in adolescents or cognitively impaired adults and the incidence is higher in infants under the age of 2 years. In most cases etiology is accidental, although bad hygiene habits are considered risk factors [2]. Clinical presentations are edema, redness up to tissue necrosis, depending on duration and entity of constriction [1, 2]. Mat Saad et al. [3] reported a unique case of HTTS in a 3-month-old baby with erosion of the middle phalanx of the third toe. We present a rare case of a HTTS with bone erosion and partial flexor tendon lesion. To the best our knowledge, these combined injuries have not previously described.

## 2. Case Report

An 8-week-old girl was admitted to the Emergency Department with a worsening swelling and redness of the right second and third toes. Four days earlier, the mother had noticed the swelling and had taken her baby to the pediatrician. Local antibiotic and bandaging were started thinking of an infection, but swelling and redness rapidly increased.

**Figure 1:**
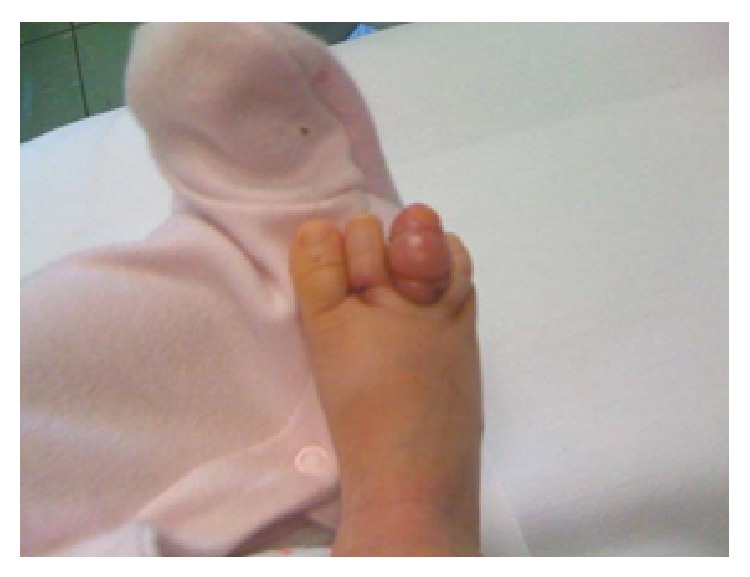
Right foot on admission. Note swelling and redness of the second and third toes.

**Figure 2:**
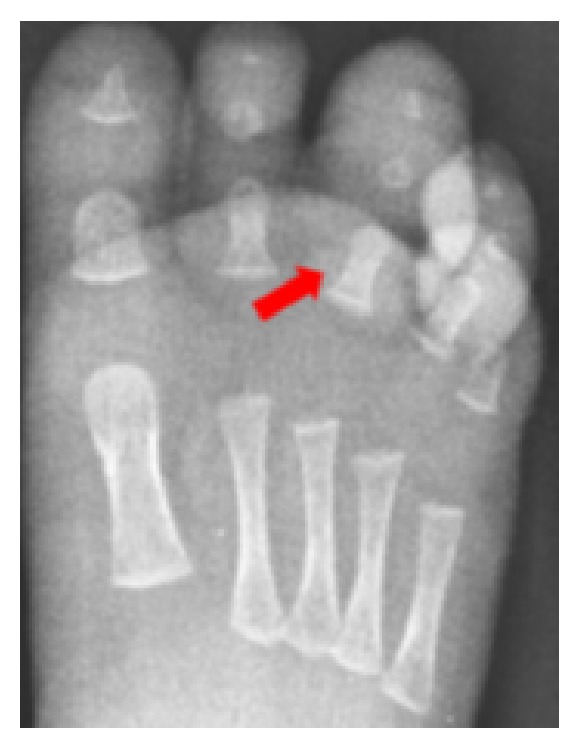
Plan X-ray showing bone erosion (arrow).

At admission to the Orthopedic Department, the patient was quiet and afebrile, with no signs of systemic involvement. No congenital deformity or trauma was reported. Inflammatory blood markers were normal. The third toe revealed significant swelling, congestion, and violet color; the second toe appeared to be edematous, with normal color (Figure 1). Capillary refilling appeared to be good in both toes. With magnifying loupes, a three-millimeter black hair was found protruding dorsally from the base of the swollen third toe. The hair was rolled around the base of the toe, but the deep constriction was invisible because of severe skin swelling. The second toe appeared less edematous with normal color; another black hair was noticed rolled at the base. Diffuse skin and deep-tissue maceration was recognized at the base of the third toe on plantar region. Surgical inspection of the wound revealed partial flexor tendon lesion and bone erosion. The hair was taken and circumferentially unwound from the third toe using a surgical forceps. It was rolled around the metatarsophalangeal joint and the interphalangeal joint. Diffuse deep-tissue maceration and ulceration prevented repairing the tendon. A second hair was noticed and unrolled from the base of the second toe. Bandaging without sutures was performed. Postoperative X-rays confirmed bone erosion crossing the proximal phalanx of the third toe (Figure 2).

**Figure 3:**
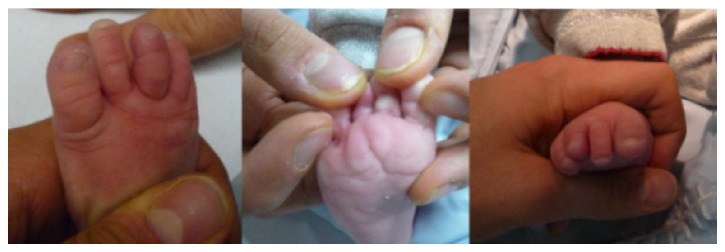
Right foot 5 weeks after treatment, demonstrating reduction of swelling and limited flexion of the third toe.

Systemic antibiotic therapy (ceftriaxone 250 mg and teicoplanin 40 mg infusion) was administered for 7 days. Swelling decreased and the wound healed satisfactorily in 5 weeks with no signs of tissue necrosis, infection, or neurovascular problems, but a decreased flexion of the third toe was evident (Figure 3).

## 3. Discussion

Hair toe tourniquet syndrome (HTTS) indicates a pediatric emergency where toe is constricted by hair or fibers causing ischemic strangulation [1, 2]. Although the first case of HTTS was seen in 1612, in modern society it is still rarely recognized [4]. The third toe is most frequently involved in 31.5% of cases, and two toes are involved in 23.5% of cases. Incidence is higher in infants under the age of 2 years, with a median age of 4 months [5].

HTTS has been recognized to be mostly an accidental injury, although the hypothesis of child abuse must be considered [2]. Many causes may be taken into account, such as the telogen effluvium in the mother's hair-loss period after pregnancy or loss of fibers using old clothes or socks [6]. Some authors have postulated that it occurs by chance, with the baby's digital movements within the loose fabric of clothing such as mittens or socks [7]. Moreover, cases have been described in babies living in extreme poverty and where bad behavior by the parents is also found [2].

The only sign of HTTS in neonates could be excessive prolonged crying, because the hair cuts the skin and can be buried under it; older infants can complain of pain and difficulty in ambulation [3]. The constriction blocks lymphatic drainage, creates local tissue edema, and reduces the venous outflow; an ischemic condition develops hour by hour. If not promptly diagnosed and treated, the constriction can lead to tissue necrosis [3].

Diagnosis of HTTS is essentially clinical. Radiographs should be taken to identify bone erosions in cases of serious and prolonged constriction [3]. Differential diagnosis of HTTS includes infections, ainhum (dactylosis spontanea), and congenital constriction band syndrome [2]. In the literature, HTTS is usually described only with swelling and circulatory suffering of toes; only one case has been reported of a bone lesion due to prolonged constriction and local tissue ulceration [3]. In our unique case, a progressive deepening of the hair into the soft tissue of the third toe produced a partial lesion of flexor tendon and bone erosion as confirmed on X-ray.

The goal of treatment for HTTS is to remove constriction. Many different techniques have been described. Some authors suggest that techniques using scalpels or needles to get under the hair tourniquet are difficult because of the swelling; the risk of damaging healthy tissues is high [8]. O'Gorman and Ratnapalan [9] used a depilatory cream to destroy such hair, concluding that this is safe and can be performed with minimal discomfort to the patient. However, this technique cannot be used if the hair fibers are deeply embedded in the edematous skin and not clearly visible; we do not suggest the use of creams if there are wounds or deep-tissue exposure. A surgical technique with dorsal peritendinous incisions of a strangulated finger has been described. Serour and Gorenstein [10] suggest a short, deep, longitudinal incision over the area of strangulation on the dorsal aspect of the toe as far as the phalanx bone, using a surgical blade. Complete trans-section and release of the constricting fibers are effected in this way. The technique is simple and allows the complete release of strangulation without risks of iatrogenic soft tissue damage. Fortunately, in our case we unwound and removed hair constriction because a small part of the hairs coming out dorsally from the toes was seen.

## 4. Conclusion

HTTS has to be considered as a cause of toe swelling and discoloration in children. An accurate search of the hair must be performed using magnifying loupes because the hair tourniquet may not be detectable to the naked eye. Early recognition and urgent treatment are mandatory to avoid potential complications.
